# A guide to the identification of metabolites in NMR-based metabonomics/metabolomics experiments

**DOI:** 10.1016/j.csbj.2016.02.005

**Published:** 2016-03-09

**Authors:** Anthony C. Dona, Michael Kyriakides, Flora Scott, Elizabeth A. Shephard, Dorsa Varshavi, Kirill Veselkov, Jeremy R. Everett

**Affiliations:** a Department of Surgery and Cancer, Faculty of Medicine, Imperial College London, SW7 2AZ, United Kingdom; b Institute of Structural and Molecular Biology, University College London, London WC1E 6BT, United Kingdom; c Medway Metabonomics Research Group, University of Greenwich, Chatham Maritime, Kent ME4 4TB, United Kingdom

**Keywords:** Nuclear magnetic resonance (NMR) spectroscopy, Metabolite identification, Molecular structure, Metabonomics, Metabolomics

## Abstract

Metabonomics/metabolomics is an important science for the understanding of biological systems and the prediction of their behaviour, through the profiling of metabolites. Two technologies are routinely used in order to analyse metabolite profiles in biological fluids: nuclear magnetic resonance (NMR) spectroscopy and mass spectrometry (MS), the latter typically with hyphenation to a chromatography system such as liquid chromatography (LC), in a configuration known as LC–MS. With both NMR and MS-based detection technologies, the identification of the metabolites in the biological sample remains a significant obstacle and bottleneck. This article provides guidance on methods for metabolite identification in biological fluids using NMR spectroscopy, and is illustrated with examples from recent studies on mice.

## Highlights

• Many simple metabolites can be identified in biofluids using 1D ^1^H NMR methods.
• Metabolites at lower levels and with more complex structures will require 2D NMR approaches for identification.
• Metabolite databases such as the HMDB are helpful for metabolite identification.
• Users need to be aware of the possibility of errors in metabolite databases.
• Metabolite identification carbon efficiency (MICE) helps measure known metabolite identification confidence.

## 1 Introduction

Metabonomics is defined as ‘the quantitative measurement of the multiparametric metabolic response of living systems to pathophysiological stimuli or genetic modification’ and is concerned with the study of the metabolic response of organisms to disease, environmental change or genetic modification [1]. The similar term metabolomics [2] was defined later and is now used interchangeably. In contrast to the interventional definition of metabonomics, metabolomics has an observational definition which is difficult if not impossible to achieve: ‘a comprehensive analysis in which all the metabolites of a biological system are identified and quantified’ [2]. In this work we will use the original term throughout. Metabonomics has many areas of application including biology and medicine [3] with new developments such as pharmacometabonomics (the ability to predict drug responses prior to drug dosing) and the more general area of predictive metabonomics, emerging recently [4], [5], [6], [7].

There are many stages to a well-designed metabonomics experiment including: 1) definition of study aims and experimental design, 2) ethical approval of the study, 3) sample collection and storage, 4) sample preparation, 5) data acquisition, 6) data quality control, 7) spectroscopic data pre-processing (for NMR data this would include zero-filling, apodisation, Fourier transform, phasing, baseline correction and referencing), 8) statistical data pre-processing including peak alignment, scaling and normalisation, 9) statistical analysis of the data to interrogate e.g. differences in metabolite profiles due to a drug treatment, 10) identification of the metabolites that are responsible for the metabolite profile differences, 11) biological/biochemical interpretation of the role of those metabolites, including pathway analysis and 12) reporting of results and deposition of the data.

Many of the metabonomics study elements above have excellent literature reviews and references available to assist effective study execution [8], [9], [10], [11], [12], [13], [14], [15], [16], [17], [18], [19], [20]. However, the identification of the key biomarkers or metabolites that are responsible for discriminating between different groups in a study (Stage 10 above) is non-trivial for both NMR [15], [21], [22], [23], [24], [25], [26], [27], [28] and MS-based [28], [29], [30], [31], [32], [33], [34] metabonomics experiments. This guide aims to provide an insight into the methodologies that can be used for NMR-based metabolite identification in the course of a metabonomics project. It is assumed that the reader is familiar with the basics of NMR spectroscopy: many excellent books on the topic are available [35], [36], [37], [38]. The focus of this guide is on the *use* of ^1^H NMR, or ^1^H NMR-detected heteronuclear 2D NMR experiments, for metabolite identification in metabonomics experiments on biological fluids.

## 2 Molecular structure information from 1D NMR spectra of metabolites

A surprising amount of information is available from a one-dimensional (1D) ^1^H NMR spectrum, including: 1) chemical shifts, 2) signal multiplicities, 3) homonuclear (^1^H - ^1^H) coupling constants, 4) heteronuclear coupling constants (typically ^14^N–^1^H or ^31^P–^1^H), 5) the first order or second-order nature of the signal, 6) the half bandwidth of the signal, 7) the integral of the signal and 8) the stability of the signal (changes in the integral with time). We will not cover: 9) spin–lattice relaxation times (T_1_s) or 10) spin–spin relaxation times (T_2_s). Whilst an appreciation of both these latter features is critical for the conduct of all NMR experiments, and differentiation of short from long T_2_s is fundamental in the Carr–Purcell–Meiboom–Gill (CPMG) spin-echo pulse sequence for plasma analysis, these features are of minor importance *per se* for metabolite identification. We will deal with each of the first 8 features in turn and see how they can be used to assist metabolite identification.

### 2.1 ^1^H NMR chemical shifts

Each chemically distinct hydrogen nucleus in each metabolite in a biological sample, such as a biofluid, will exhibit an NMR signal at a characteristic resonance frequency, which is measured as a chemical shift relative to a standard compound. For example, in metabonomics studies of urine, it is common to add a reference material such as 3-(trimethylsilyl)-2,2′,3,3′-tetradeuteropropionic acid (usually abbreviated to TSP) or deuterated forms of 4,4-dimethyl-4-silapentane-1-sulfonic acid (DSS) or its sodium salt, and define the chemical shift of the TSP or DSS methyl resonances as 0 ppm. Our preference is to use TSP as the reference material in biofluids without significant protein concentrations. The normal reference material for NMR spectroscopy in organic chemistry, tetramethylsilane (TMS) is rarely used in metabonomics studies, as it is insoluble in aqueous solutions.

The exact chemical shift of the NMR signal of a hydrogen nucleus in a metabolite is independent of the applied field strength, is highly reproducible and precisely characteristic of that nucleus, in that metabolite, in the particular matrix conditions. For biofluids such as blood plasma or serum, where DSS or TSP may become bound to macromolecule components, it is common to reference the spectra to the H1’ anomeric proton of the alpha anomer of glucose at 5.233 ppm, to avoid variation in reference intensity and position due to binding [39]. However, care must be taken with temperature control as this signal has high temperature sensitivity and indeed, has been used as an NMR thermometer [40].

**Fig. 1:**
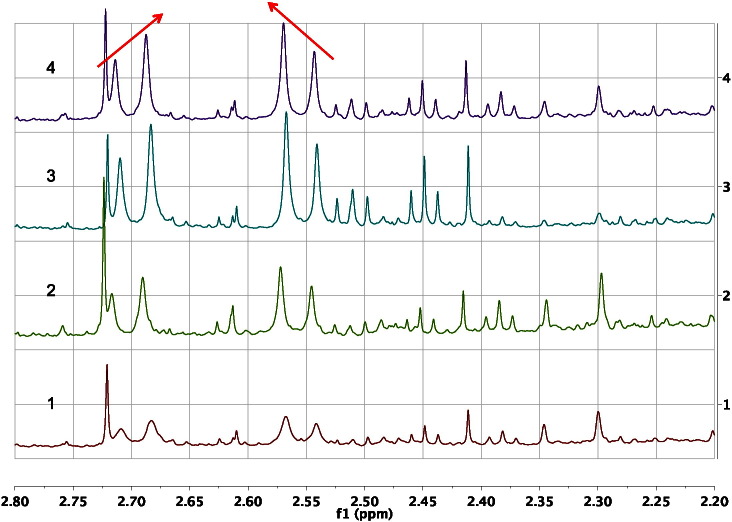
an expansion of the 600 MHz ^1^H NMR spectrum of the urine of four, 30-week-old, male C57BL/6 mice, in the region of the doublet signals of citrate at ca 2.70 and 2.56 ppm. Even though the urine is buffered to pH 7.4, there are differences in the chemical shifts of the citrate signals between the four urine samples and noticeable differences also in half bandwidth, with the signals of mouse 1 (bottom spectrum) being especially broadened. The ‘roofing’ of the doublet citrate signals towards one another is illustrated by the arrows above the citrate resonances of mouse 4. See Section 2.6 on 2nd order effects.

When comparing the experimental ^1^H NMR chemical shifts of hydrogens in metabolites in intact biofluids with those of the corresponding pure reference standards in aqueous solution, it is usual for values to agree within 0.03 ppm. One of the strengths of NMR spectroscopy is that the chemical shifts are exquisitely sensitive to structural and environmental change. Indeed, sensitivity of chemical shifts to pH change can be used to distinguish or identify metabolites, especially those containing ionisable functional groups [41]. Whilst this is an excellent feature in terms of decreasing the likelihood of two similar molecules having identical ^1^H NMR spectra, it does mean that for some metabolites, environmental change can have a significant effect on the spectra, including the ^1^H NMR chemical shifts. A classic case of this sensitivity to the environment occurs for the diastereotopic methylene hydrogens in citric acid. Changes in pH between samples will alter the ionisation of the carboxylate groups in citric acid and thus affect the chemical shifts of the methylene hydrogens. In addition, it is well known [3] that citric acid can chelate metal ions such as calcium, magnesium and sodium. Thus, even if biofluid samples are buffered effectively to a constant pH, changes in metal ion concentrations between samples, which are not readily apparent by ^1^H NMR, may have a significant effect on the chemical shifts and the half bandwidths of the signals of the methylene hydrogens of citric acid and also any other metabolites with similar properties. This effect is observed in Fig. 1.

Many general resources are available which correlate the relationships between chemical structure and NMR chemical shifts [36], [42], including web resources [43], whilst more specific metabonomics-focused databases are covered in Section 4.3 below. As for ^13^C NMR chemical shifts (see Section 2.2 below), it is also possible to calculate ^1^H NMR shifts, especially in discrete series [43].

### 2.2 ^13^C NMR chemical shifts

Most metabonomics experiments are conducted with ^1^H NMR detection. However, the 2D ^13^C, ^1^H HSQC NMR (Section 3.5 below) and 2D ^13^C, ^1^H HMBC NMR (Section 3.6) experiments which correlate ^1^H NMR chemical shifts with ^13^C NMR chemical shifts over 1-bond (HSQC) or 2 to 3 bonds (HMBC) are very important for metabolite identification, as they enable the determination of the ^13^C NMR chemical shifts of metabolites, so an appreciation of the nature of ^13^C NMR chemical shifts is required. One key feature of ^13^C NMR chemical shifts is their much larger range of values compared with ^1^H NMR chemical shifts. For common metabolites ^13^C NMR chemical shifts occupy a huge range of values from ca 10 ppm for methyl carbons such as C4 in butanone to ca 222 ppm for the ketone (C2) carbon in the same molecule. Thus the range of ^13^C NMR chemical shifts is ca 20 times that of ^1^H NMR and this is the reason that their measurement is so important in metabolite identification: they are much more sensitive to small changes, or more remote changes in molecular structure, including stereoisomerism, than ^1^H NMR chemical shifts.

For simple molecules ^13^C NMR chemical shifts can be calculated by hand using simple additivity tables.[44] For example in simple substituted benzenes such as para-cresol (1-hydroxy-4-methylbenzene), the ^13^C NMR chemical shifts of all of the carbons can be calculated by adding the known substituent effects of hydroxyl and methyl groups [44] to the chemical shift of benzene in an additive fashion.

**Figure:**
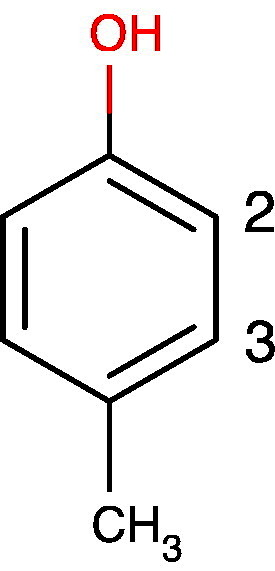

The substituent parameters for an hydroxyl group added to a benzene ring are + 26.9 (ipso), − 12.7 (ortho), + 1.4 (meta) and − 7.3 ppm (para position). For a methyl group the corresponding parameters are + 9.3 (ipso), + 0.8 (ortho), 0.0 (meta) and − 2.9 ppm (para) [44]. The accepted ^13^C NMR chemical shift of benzene is 128.5 ppm. Even if the molecule para-cresol was not in a metabolite database, we could calculate the ^13^C NMR shifts with some degree of precision. For C2 and C3 the calculated shifts would be as follows:

$$\delta_{C2}=128.5–12.7\;\left(\mathrm{OH}\;\text{ortho}\right)+0\;\left(\text{methyl},\text{meta}\right)=115.8\;\mathrm{ppm}$$

$$\delta_{C3}=128.5+1.4\;\left(\mathrm{OH}\;\text{meta}\right)+0.8\;\left(\text{methyl},\text{ortho}\right)=130.7\;\mathrm{ppm}$$

For comparison, the actual values in the HMDB [45] for para-cresol, HMDB01858, in water at pH 7.0 are 117.9 and 132.8 ppm for C2 and C3 respectively. Modern NMR data processing software such as MNova [46] possesses more sophisticated ^1^H, ^13^C, and multinuclear NMR chemical shift calculation and prediction algorithms. The algorithm in MNova 10.0.0 predicted shifts of 117.6 and 130.0 ppm for C2 and C3 respectively, a very good fit to the real data for C2, but not quite as good as the simple hand calculation for C3. MarvinSketch v 6.1.1 from ChemAxon [47] also has ^1^H and ^13^C NMR chemical shift calculation capabilities and it gave 115.3 and 130.0 ppm for C2 and C3 respectively. Calculations such as these can be useful when information about metabolites of interest is not in the existing databases: a common occurrence. However, users must be aware that these calculations are approximate, with precision varying according to the complexity of the metabolite and the relationship of the structure of the metabolite to the molecules in the prediction calculation database, or to those used to derive the substituent tables. In general a precision of better than +/− 5 ppm is usually achieved for ^13^C resonances.

It is beyond the scope of this guide to discuss factors that influence ^13^C NMR shifts in any detail. However, the key factors include: hybridisation of the carbon atom (sp3, sp2 or sp. hybridised), inductive substituent effects and mesomeric effects [43], [48], [49], [50], [51], [52].

### 2.3 ^1^H NMR multiplicities

The multiplicity is the pattern of peaks that is observed for a particular hydrogen signal in the ^1^H NMR spectrum. In a first order ^1^H NMR spectrum, the frequency difference between the resonances of coupled hydrogens is large (≫ 10 times) relative to the value of the coupling constant between them. In those circumstances, the signals exhibit first order coupling patterns, which obey an n + 1 splitting rule, where n is the number of equivalent coupling partners. For instance, methyl groups such as those of lactic acid which couple with one hydrogen on an adjacent carbon via a homonuclear, 3-bond vicinal coupling, ^3^J_H,H_, will be split into a doublet signal (1 + 1 = 2). Correspondingly, the signal of the lactate methyne CH proton will be split into a 4-line quartet due to the interaction with the 3 equivalent methyl hydrogens (3 + 1 = 4). The intensity ratios of these multiplet signals follow Pascal's triangle [35], being 1:1, 1:2:1, and 1:3:3:1 for a doublet, triplet and quartet respectively. An example of a 1:2:1 triplet from one of the methylene CH_2_ groups in 2-oxoglutaric acid is clearly observed at 2.45 ppm in the ^1^H NMR spectra of the urines of the mice in Fig. 1.

**Fig. 2:**
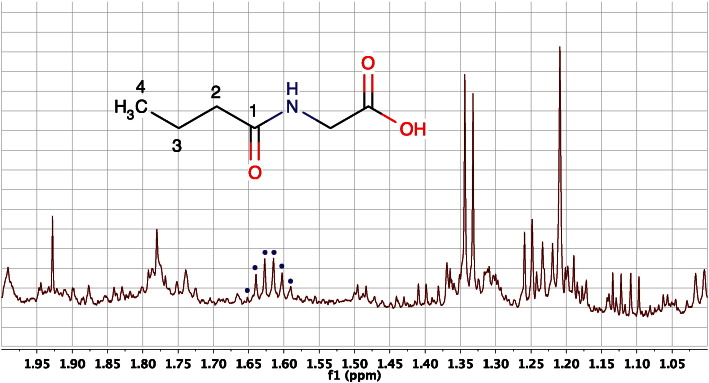
An expansion of the 600 MHz ^1^H NMR spectrum of the urine of a male, 15-week-old, C57BL/6 mouse, in the region of the signal from the CH_2_–3 methylene protons of *N-*butyrylglycine at ca 1.62 ppm (dots). This pseudo-sextet signal is actually a triplet of quartets with the two ^3^J_H,H_ couplings being almost equal in magnitude (7.4 and 7.5 Hz) resulting in overlap of many of the lines. The molecular structure of the metabolite is superimposed.

If a particular hydrogen is coupled to more than one group of hydrogens, then more complex coupling patterns or multiplicities are observed. For instance, the CH_2_–3 methylene signal from the butyryl chain of *N*-butyrylglycine resonates as a triplet of quartets as it is coupled to both the terminal CH_3_–4 protons and the CH_2_–2 protons adjacent to the C1 amide carbon. If the coupling constants involved were non-equal then up to 12 lines could be observed (4 × 3). However, in this case, the CH_3_–4 to CH_2_–3 coupling constant (7.4 Hz) is almost equal to the CH_2_–2 to CH_2_–3 coupling constant (7.5 Hz) and the C3 methylene signal resonates as a pseudo-sextet due to signal overlap (Fig. 2).

The analysis of signal multiplicities, simple and complex [36], [43], is important for the identification of metabolites. Multiplicity-editing spin-echo NMR experiments can also be used to distinguish between signals with different multiplicities and this can be helpful in metabolite identification. A good example is the use of spin-echo ^1^H NMR in the identification of novel penicillin metabolites, where the characteristic singlet signals of the penicillin gem-dimethyl groups can be easily identified by Hahn spin-echo methods [53], [54]. Many spectra do not obey first order requirements however and two main consequences arise from this; multiplicity intensities may be distorted, or in extreme cases additional lines may occur in the multiplets: see Section 2.6 below.

### 2.4 Homonuclear ^1^H, ^1^H coupling

Scalar coupling can occur between all non-equivalent hydrogen atoms in a metabolite. The key requirement here is magnetic non-equivalence. Hydrogens that are equivalent by molecular symmetry, such as the methyne hydrogens in tartaric acid, or equivalent by virtue of fast rotation, such as those of methyl hydrogens, will not show scalar coupling between themselves. Indeed the two methyne hydrogens of 2R, 3R-tartaric acid resonate as a characteristic, sharp singlet at 4.34 ppm in urine.



The detection of the presence of scalar coupling between two hydrogens in a metabolite is very important in metabolite identification, as the magnitudes of the coupling constants are characteristic of the electronic pathway between the two hydrogens or groups of hydrogens. Scalar coupling is transmitted via the bonding electrons in the metabolites and drops off in magnitude as the number of bonds between the hydrogens increases. Most of the homonuclear scalar couplings observed in metabolites will be two-bond geminal couplings (^2^J_H,H_) between hydrogens on the same carbon, or three-bond, vicinal couplings (^3^J_H,H_) between hydrogens on adjacent carbons in a metabolite. In general, 2-bond geminal couplings are larger in magnitude than 3-bond, vicinal couplings. However, geminal couplings are affected by the hybridisation of the carbon atom and by the electronegativity of substituents, and in some alkenes, such as R_1_R_2_C = CH_2_ the ^2^J_H,H_ value for the terminal = CH_2_ will be close to 0 Hz. In passing, we should note that most geminal couplings are negative in sign and most vicinal couplings are positive, but this is not relevant for most analyses and we will ignore this feature henceforth. The large magnitude of geminal couplings in sp^3^CH_2_ groups is well illustrated by the spectrum of citrate shown in Fig. 1, where the geminal ^2^J_H,H_ coupling is ca 16.2 Hz. By contrast, the 3-bond, vicinal coupling between the C3–CH_2_ group and its adjacent methyl and methylene group neighbours in *N-*butyrylglycine is ca 7.4 and 7.5 Hz respectively (Fig. 2). These ^3^J_H,H_ values are smaller and are typical of the values for free-rotating aliphatic moieties.

The values of vicinal couplings are particularly sensitive to stereochemistry in relatively rigid systems and this is well illustrated by metabolites such as D-glucose, which exists as two anomers in slow exchange with one another, so that separate signals are observed for each anomer.

**Figure:**
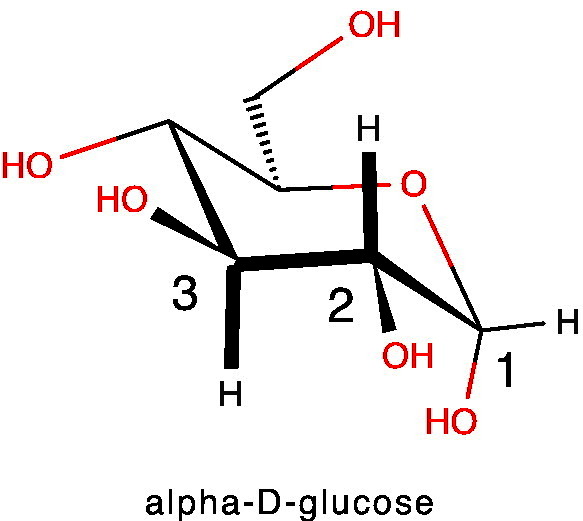

The anomeric proton at C1 in the alpha anomer is in an equatorial position on the 6-membered pyranose ring and has a modest ^3^J_H,H_ coupling of ca 3.7 Hz to the axial H-2 (equatorial–axial coupling). By contrast, the coupling between H2 and H3 (both axial) has a value ^3^J_H,H_ ca 9.8 Hz because this is a favoured, di-axial coupling. Thus the magnitude of coupling constants can give information on the type of coupling and the stereochemistry of the interacting hydrogens. In addition to this, the values of coupling constants are affected by the electronegativity of groups in their vicinity, due to their impact on the electrons that transmit the coupling [43].

**Fig. 3:**
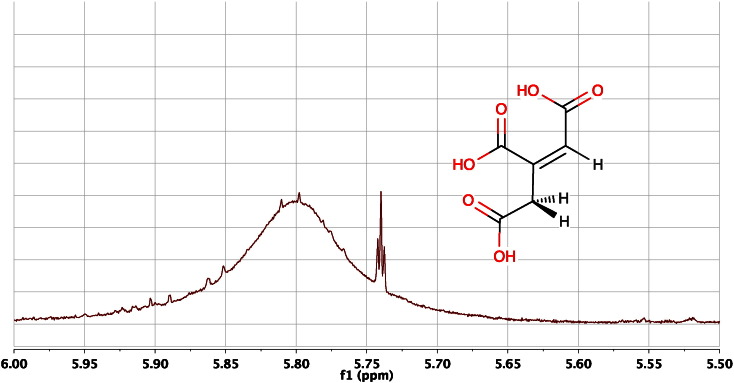
An expansion of the 600 MHz ^1^H NMR spectrum of the urine of pooled, male C57BL/6 mice at 15 weeks of age, in the region of the signal from the olefinic proton of *cis-*aconitic acid at ca 5.74 ppm. The signal is a triplet due to a long-range, 4-bond, ^4^J_H,H_ coupling of ca 1.4 Hz to the two equivalent methylene hydrogens. The 1:2:1 nature of the triplet is clear, even though it is superimposed upon the very broad signal from urea at ca 5.80 ppm.

If ^1^H NMR spectra are acquired with good spectral resolution, good digital resolution and good lineshape, it is possible to observe 4-bond, 5-bond and even 6-bond hydrogen-to-hydrogen couplings, ^4^J_H,H_, ^5^J_H,H_ and ^6^J_H,H_, in biofluids. For example, in cis-aconitic acid, it is usual to observe the olefinic proton at ca 5.74 as a triplet with ^4^J_H,H_ ca 1.4 Hz due to long-range, 4-bond coupling to the equivalent methylene CH_2_ hydrogens across the double bond (Fig. 3).

In the trans-aconitic acid isomer, the olefinic proton at 6.60 ppm is a triplet with a smaller ^4^J_H,H_ ca 0.8 Hz coupling. Note the enormous sensitivity of the chemical shift to the geometry of the double bond: the olefinic proton shifts nearly 0.9 ppm just from the change of double-bond geometry, and the change in the coupling value for ^4^J_H,H_ is also diagnostic.

### 2.5 Heteronuclear ^1^H, X coupling

**Fig. 4:**
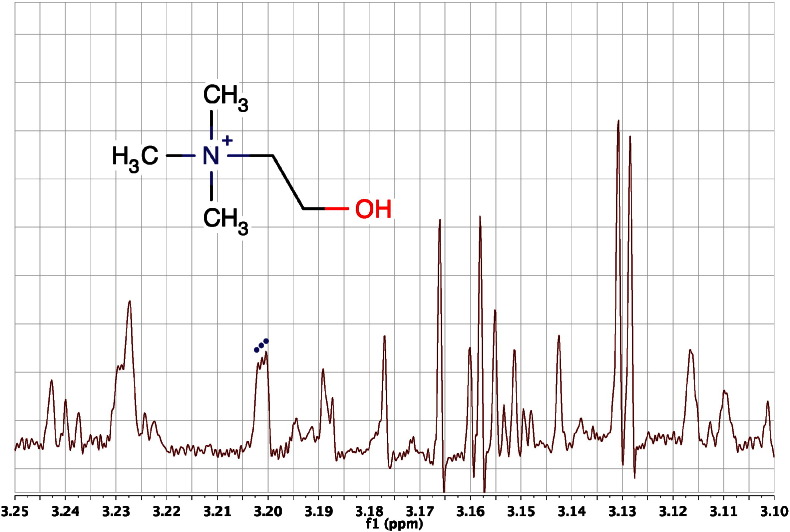
An expansion of the 600 MHz ^1^H NMR spectrum of the pooled urine of male C57BL/6 mice at 15 weeks of age, in the region of the signal from the methyl protons of choline (structure superimposed) at ca 3.20 ppm. The signal is a 1:1:1 triplet (dots) due to a 2-bond coupling of ca 0.6 Hz to the ^14^N nucleus. Interestingly, the well-resolved doublet at ca 3.13 ppm is due to the methylene protons of cis-aconitic acid with ^4^J_H,H_ coupling of ca 1.4 Hz (see also Fig. 3). The spectrum has been zero filled to 131,072 points and resolution enhanced by Gaussian multiplication, prior to Fourier transformation.

These couplings are less common but will occur in phosphorous-containing metabolites such as adenosine monophosphate, where the presence of the NMR-active, 100% abundant, spin I = 1/2, ^31^P isotope will give rise to additional 3-bond and 4-bond ^3^J_P,H_ and ^4^J_P,H_ couplings to the ribose ring protons, that are highly diagnostic [55]. Another less-commonly observed heteronuclear coupling in metabolites is due to the 99.6% natural abundance ^14^N isotope which is NMR-active but quadrupolar, with spin quantum number I = 1. Due to quadrupolar relaxation, couplings to ^14^N are not often observed, but in a symmetrical environment, the effects of quadrupolar relaxation are reduced and small couplings may be observed and these can also be critical for metabolite identification. For instance, in choline (HMDB00097), the almost symmetrical environment around the nitrogen allows the observation of a small ^2^J_N,H_ coupling of ca 0.6 Hz (1:1:1 triplet due to spin quantum number I = 1) to the methyl hydrogens due to 2-bond coupling to the ^14^N. So, in this unusual case, the methyl signal is a narrow triplet instead of the expected singlet (Fig. 4).

### 2.6 Second-order effects and strong coupling in ^1^H NMR spectra

As the frequency separation in Hertz between coupled ^1^H NMR signals decreases to less that ca 10 times the value of the coupling constant between them, distortions to expected multiplet peak intensities start to occur in the spectra. The spins are said to exhibit ‘strong coupling’, or to be in a second-order system. The spectra take on appearances that are different from those of systems that exhibit ‘weak coupling’ or are in first-order systems. Rather than being a problem, this is actually an aid to spectral interpretation and metabolite identification, as follows. In the simple case of two, non-equivalent hydrogen atoms coupling with one another, the intensity distortion is such that the doublets slope towards one another in an effect called ‘roofing’. This is well illustrated in the spectra of the two, non-equivalent methylene protons in citric acid shown in Fig. 1. The chemical shift difference between the resonances at 2.70 and 2.56 ppm is 0.14 ppm, which equates to ca 84 Hz at 600 MHz operating frequency. The ^2^J_H,H_ coupling is ca 16.2 Hz and therefore the ratio of the frequency separation to the coupling constant is 84/16.2 = 5.2. This two-hydrogen spin system is formally designated AB: the two letters indicate that there are two distinct spins or hydrogen atoms involved in the coupling system; the closeness of the letters in the alphabet indicates that their chemical shifts are close in frequency. The roofing of the signals is clear to see in Fig. 1 and provides a way, without using 2D COSY NMR or any decoupling techniques, to determine that these hydrogens are coupled to one another; an important and often overlooked benefit of this feature. In a two-spin system that is first order, the nomenclature would be AX instead of AB to indicate that the two hydrogens are widely separated in chemical shifts, relative to the size of their mutual coupling.

**Fig. 5:**
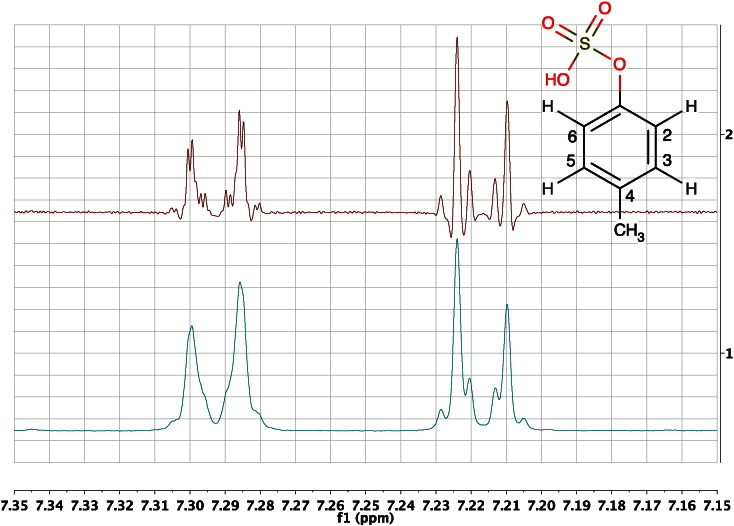
Two versions of the 600 MHz ^1^H NMR spectrum of an authentic sample of the metabolite *para*-cresol sulphate in deuterated phosphate buffer at pH 7.4, in the region of the signals from the aromatic hydrogens: 1) with a standard 0.3 Hz line-broadening and 2) resolution-enhanced using a Lorentzian to Gaussian transformation. The signal of the H2, H6 protons appears as a complex, second-order multiplet at ca 7.22 ppm, instead of a first-order doublet. The signal of the H3, H5 protons at ca 7.29 ppm displays additional complexity due to coupling to the methyl protons via a 4-bond coupling, in addition to the extra lines, clearly visible in this second-order system.

If the spin system is more complex, or the ratio of signal frequency separation to coupling constant becomes much smaller, the intensity distortions can become more significant and in extreme cases involving three spins or more, additional lines are seen in the resonances which are not always interpretable by first order analysis. This effect is commonly observed in the NMR signals for the aromatic hydrogens in symmetrically substituted benzene rings (Fig. 5).

**Fig. 6:**
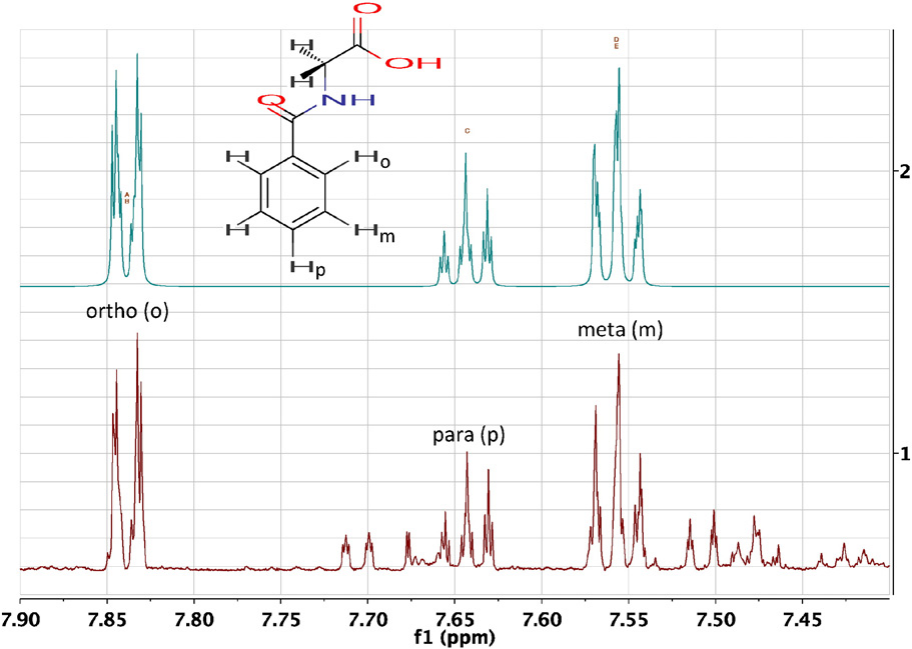
1) The 600 MHz ^1^H NMR spectrum of the urine of a 30-week-old, male, flavin mono-oxygenase 5 (FMO5) knockout mouse [61] in the region of the aromatic signals from hippuric acid (structure inset). The spectrum is resolution enhanced by Gaussian multiplication. 2) A spin simulation of the aromatic signals from hippuric acid using the MNova spin simulation function. A good approximation to the complex, second-order signals was obtained. The complexity of the two ortho and two meta hydrogen signals is due to the fact that whilst these hydrogens are chemically equivalent (within each pair), they are magnetically non-equivalent and are part of a five hydrogen AA’BB’M spin system (see Section 2.6). Signals from 3-indoxyl sulphate and other metabolites are present in the real spectrum (1).

For metabolites such as *para*-cresol sulphate, the phenomenon of magnetic non-equivalence appears [37]. The hydrogens on C2 and C6 are chemically equivalent by symmetry, as are those on C3 and C5. However, these pairs of hydrogens are NOT magnetically equivalent. The reason for this is as follows: H2 is *ortho* to H3 and has a 3-bond coupling to it. By contrast, the chemically equivalent proton H6 is *ortho* to H5 and *para* to H3. Thus, in terms of their nuclear magnetic interactions, these hydrogens are non-equivalent and this has consequences. The frequency difference between the signals of H2 and H6 is 0 Hz by definition and they are coupled by a favourable, long-range ‘W’ coupling over 4-bonds. Thus, the frequency separation to coupling ratio is 0, no matter what the value of the coupling constant and the resultant spectra are second-order [37]. This spin system is designated AA’BB’, where A and A’ represent H2 and H6 and the apostrophe signifies a chemically equivalent but magnetically non-equivalent nucleus. B and B’ are H3 and H5 and the closeness of the letters in the alphabet is deliberate and signifies the closeness of the chemical shifts of these two groups of spins. In these extreme cases, additional lines appear in the spectra and the resonance patterns may not be readily interpretable by first order analysis. Instead of a simple pair of doublets, as might be expected, a complex pattern appears (Fig. 5). Typically, a computational, spin simulation program is used to calculate the spectrum and this is now a routine procedure. An important point to appreciate is that it may not be straightforward to extract chemical shifts or coupling constants from second-order spectra without spin simulation: see Section 2.10 and Fig. 6.

### 2.7 The half bandwidth of NMR signals

Another feature that provides information on the structure and the dynamics of metabolites is the half bandwidth of their signals. The half bandwidth, Δν_1/2_ of a signal is related to the real spin–spin relaxation time of the hydrogen giving rise to that signal according to Eq. (1):

(1) $$\Delta \nu_{1/2}=1/\pi .T_{2}*$$...

where T_2_* is the real spin–spin relaxation time that takes into account underlying molecular relaxation processes, plus the effect of field inhomogeneities and the influence of factors such as the presence of paramagnetic species (including dissolved oxygen gas) in the sample. T_2_* can be shortened by interaction with quadrupolar spins, such as ^14^N and by chemical exchange. In the case of chemical exchange between two forms of a metabolite, A and B, the lifetime of a spin in species A is necessarily limited to the lifetime of species A, as a maximum. Exchange-broadening of the signals will occur when the exchange rate in Hertz between forms A and B is of the same magnitude as the chemical shift difference in Hertz between the corresponding hydrogens in A and B. The broadening effects of exchange with water, quadrupolar relaxation and unresolved couplings to ^14^N can be quite large, as can be seen in Fig. 3, where the hydrogen signal from urea has a half bandwidth of ca 50 Hz, in contrast to the much narrower linewidth of the olefinic proton in cis-aconitic acid, where all of these effects are absent and consequently the non-exchanging hydrogens have much larger T_2_* values_._

### 2.8 The integral of NMR signals

When NMR experiments are run with sufficient delay times in between the acquisition of each successive free induction decay, the nuclei under observation will enjoy close to full spin–lattice relaxation. Under these conditions, the signals will not be partially saturated [35], and the area of a methyl (CH_3_) signal in a metabolite in a biofluid will be precisely three times that of a methyne (CH) signal in the same metabolite in the same sample. NMR spectroscopy is thus an inherently quantitative technique and this is a huge advantage for the conduct of metabonomics experiments. It should be noted however that most NMR-based metabolic profiling experiments do not achieve full relaxation with the delay times typically used. Even so, the situation is in stark contrast to MS-based profiling, where the intensities of signals from metabolites may be significantly suppressed or enhanced by the presence of other metabolites in the sample [56] and internal reference standards are required in order to achieve quantitation.

Quantifying the level of a metabolite in a biological fluid such as urine, by ^1^H NMR spectroscopy, can be very difficult, because of spectral crowding and spectral overlap, and great care is required either with line fitting or direct integration quantification approaches. However, when careful approaches are taken, the analytical precision of the methodology is high [12], [57], [58] and this is critical for the statistical analysis of the data and the reliable discovery of discriminating biomarkers: see Section 4.1.

### 2.9 The stability of NMR signals

Generally, the metabolic profile of a biological fluid is stable over a significant period of time at room temperature, and certainly stable enough for the acquisition of routine 1D and 2D ^1^H NMR data. However, there are exceptions. Some biological fluids are inherently unstable. A good example of this is human seminal fluid, where, post-ejaculation, enzymatic reactions take place that cause the biochemical transformation of some metabolites [59]. In addition, if a sample such as animal urine, has been in contact with animal faeces at any stage, it will be microbiologically contaminated and potentially unstable. Bacterial growth in a urine sample, for instance, will result in the transformation of certain metabolites into new products, as the bacteria scavenge the biofluid for fuel sources. It is common practice to add anti-bacterial agents such as sodium azide [8], [16] to inhibit the growth of the bacteria. However, in our experience, even in the presence of sodium azide at 9 mM, bacterially-mediated metabolite transformations can still occur in mouse urine if kept at room temperature for extended periods, and hence, signals will be unstable over time: the signals of fermentation substrates will decrease, whereas those of products will increase. See Section 4.6, Biochemical Transformation and *In Vitro* Fermentation of Biofluids to Aid Metabolite Identification. A major improvement in this area has occurred with the development of cooled sample changers, such as the SampleJet system from Bruker Corporation (Billerica, Massachusetts, USA), that keeps queued samples at 4 C prior to their insertion into the NMR magnet, thus minimising sample instability.

### 2.10 Interpretation of 1D ^1^H NMR spectra and metabolite identification

Metabolites that are present at relatively high concentrations or that have distinctive signals in relatively uncrowded spectral regions can be identified by inspection from a simple 1D ^1^H NMR spectrum. This can be done manually by the spectroscopist interpreting the data, or with the assistance of software such as Chenomx NMR Suite (Chenomx, Edmonton, Canada), which has the advantage of a database of standard metabolite spectra at a variety of magnetic field strengths and a variety of pH values [60]. Obvious metabolites include citric acid (see Fig. 1) where the (somewhat variable) chemical shifts and large ‘roofed’ geminal couplings of the methylene protons are unmistakeable. Another easily identifiable metabolite is hippuric acid, whose second-order aromatic proton resonances between 7.9 and 7.4 ppm provide an unmistakable ‘fingerprint’ for identification (Fig. 6).

Certain other metabolites have distinctive singlet signals at characteristic chemical shifts, such as the methyl hydrogens of methylamine, dimethylamine and trimethylamine at ca 2.61, 2.73 and 2.88 ppm respectively. However, little information is present in the 1D ^1^H NMR spectrum of these metabolites: just one singlet resonance. Hence, it is advisable to check the assignments of these types of resonances using a 2D ^13^C, ^1^H HSQC experiment to verify that the methyl carbons have the expected chemical shifts of ca 27.7, 37.6 and 47.6 respectively for methylamine, dimethylamine and trimethylamine. Note the uniform ca 10 ppm increase in methyl carbon chemical shift as each methyl group is added, due to the additive, two-bond or beta substituent effect.

The identification of metabolites present at relatively low levels, or that have signals that are partially or completely overlapped, will be difficult by 1D NMR methods and the use of two-dimensional NMR spectroscopic methods is required. In Section 4.7, we will review how much information is required in order to consider the identification of a known metabolite confident.

## 3 Molecular structure information from two-dimensional (2D) NMR spectroscopy

### 3.1 Introduction to 2D NMR spectroscopy

In a 1D NMR spectrum the NMR signals are acquired as a function of a single time variable (t2) in a free induction decay (FID), typically over 65,536 data points at a ^1^H frequency of 600 MHz. This FID arises from the induction of an electric current in the receiver coils of the NMR probe by the excited nuclear magnetisations: there is no emission event detected in NMR. Fourier transformation of this FID gives rise to the conventional 1D ^1^H NMR spectrum in which NMR signal intensity (y-axis) is plotted as a function of chemical shift (x-axis). By contrast in a 2D NMR experiment, a second time dimension (t1) is artificially created by the deliberate incrementing of a time delay, known as the evolution time, between two of the radiofrequency pulses in the pulse sequence used. An FID is collected for each of m values of the evolution time, such that at the end of the experiment, m x FIDs have been collected, each containing n data points. Double Fourier transformation of this data set over both t2 and t1 results in a single 2D NMR spectrum in which signal intensity (z-axis) is plotted as a function of two orthogonal signal frequency axes; f2 and f1 corresponding to t2 and t1 in the time domain (x and y respectively). The spectra are typically displayed as contour plots where signal intensity is represented by contour lines, in much the same way that the heights of mountains and hills are represented on maps.

We shall not go into the details of the design of the 2D NMR pulse sequences, nor the analysis of how those pulse sequences give the resulting spectra, as many excellent reference works are available in this area [35], [37].

### 3.2 2D ^1^H J-resolved (JRES) NMR spectroscopy

The 2D ^1^H J-Resolved NMR Spectroscopy (JRES) experiment is one of the simplest 2D NMR experiments and one of the most useful for the analysis of the complex ^1^H NMR spectra of biological fluids [21], [22], [62]. The experimental radiofrequency pulse sequence is simply: RD - 90^0^_H_ - t1/2 - 180^0^_H_ - t1/2 - FID, where RD is a relaxation delay. The second proton pulse (180^0^_H_) occurs in the middle of the incremented evolution time (t1). In the resulting 2D ^1^H NMR JRES spectrum, the chemical shifts run along the first frequency dimension, f2, as normal, and homonuclear coupling constants are modulated (spread out) across a second frequency dimension, f1. For simple, first order spin systems, no new signals are created: the existing signals are just spread out across two frequency dimensions instead of one. This has a tremendous effect in reducing signal overlap in crowded spectral regions. The spectra are typically tilted by 45^0^ so that all the signals of a homonuclear multiplet appear at the exact same chemical shift. The projection of the 2D spectrum onto the chemical shift dimension, f2, is then effectively a broadband proton-decoupled proton NMR spectrum, in which each ^1^H resonance is a singlet. It is important to note that heteronuclear couplings are unaffected by the ^1^H 180^0^ pulse in the 2D ^1^H NMR JRES experiment and these are *not* modulated across the second dimension of the 2D spectrum [55].

**Fig. 7:**
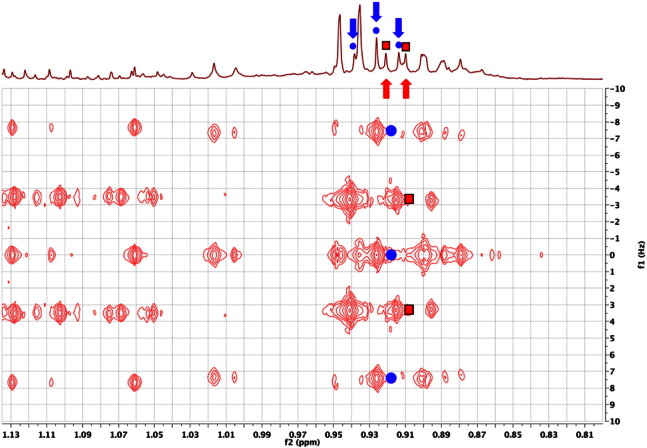
The low frequency region of the 600 MHz 2D ^1^H J-resolved NMR spectrum of the urine of a male, 30-week-old, FMO5 knockout mouse [61] displayed as a contour plot underneath the corresponding 1D ^1^H NMR spectrum. The overlapping signals from the triplet methyl group of *N*-butyrylglycine (0.926 ppm, three blue circles and downward arrows) and the doublet methyl group of isovaleric acid (0.916 ppm, two red squares and upward arrows) are completely resolved in the 2D JRES NMR spectrum. The spectrum is tilted by 45^0^, so that all the signals of each multiplet appear at the same chemical shift, and it is symmetrised.

The tremendous improvement in signal resolution by spreading the NMR signals out across a second dimension is clearly illustrated in Fig. 7. In the 1D ^1^H NMR spectrum of the urine of an FMO5 knockout mouse [61], the triplet methyl signal for *N*-butyrylglycine (three blue circles at 0.926 ppm) is overlapped with the doublet methyl signal for isovaleric acid (two red squares at 0.916 ppm). By contrast in the 2D ^1^H JRES NMR spectrum, these signals are completely resolved from one another.

**Fig. 8:**
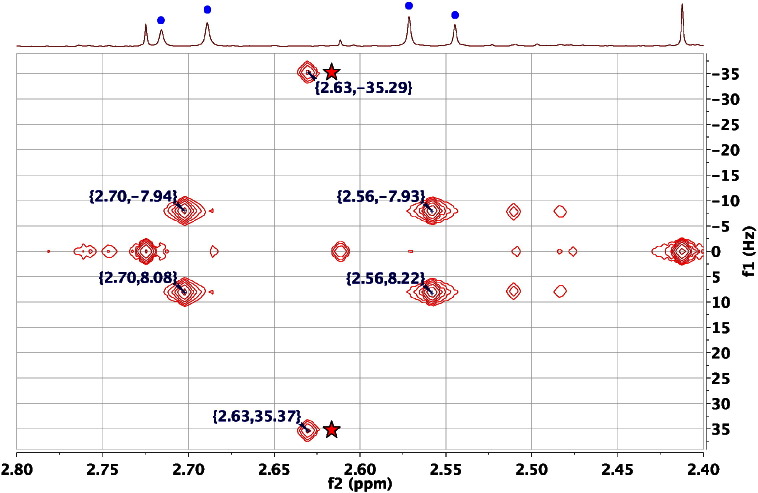
An expansion of the 600 MHz 2D ^1^H J-resolved NMR spectrum of the urine of a male, 30-week-old, FMO5 knockout mouse [61] in the region of the AB resonances from citric acid at ca 2.70 and ca 2.56 ppm (four dots in 1D spectrum), displayed as a contour plot underneath the corresponding 1D ^1^H NMR spectrum. The spectrum is tilted by 45^0^, so that all the signals of each multiplet appear at the same chemical shift, and symmetrised. The signals labelled with stars, appearing at ca 2.63 ppm, exactly in between the shifts of the two citrate signals are 2nd order effects caused by the mixing of transitions between the A and B spins by the 180^0^ pulse, in the presence of strong coupling. As is clear from the 1D ^1^H NMR spectrum, there are no real signals at 2.63 ppm!

The simple interpretation of 2D ^1^H JRES NMR spectra only applies for first order systems in which there is weak coupling. If strong coupling exists (a second-order system) then artefacts can appear in the spectra [63]. This occurs because in a strongly coupled system the second ^1^H pulse (a 180^0^ or π pulse) will cause not just the modulation of the signals of a homonuclear-coupled spin across the second dimension, according to the size of its spin couplings, it will also cause the mixing of the transitions or signals between coupled spins, such as would normally occur in a chemical shift correlation experiment such as COSY (via the second 90^0^ pulse). Thus, in a simple two hydrogen AB spin system such as citric acid, the two A transitions (doublet) become mixed with the two B transitions and in a tilted 2D ^1^H JRES NMR spectrum, signals appear in the 2D spectrum at chemical shifts where there are no hydrogens! It is very important to recognise these ‘artefacts’ in order to avoid mis-assigning the spectra to non-existent metabolites with unreal J values! Fig. 8 shows an example of this feature for citric acid itself: the 2nd order signals in the 2D ^1^H JRES NMR spectra are marked with stars.

Awareness of the origin of these signals allows chemical shift correlation information to be extracted from the 2D ^1^H JRES NMR spectrum, so these artefacts can have real utility in spectral assignment and metabolite structure elucidation!

**Fig. 9:**
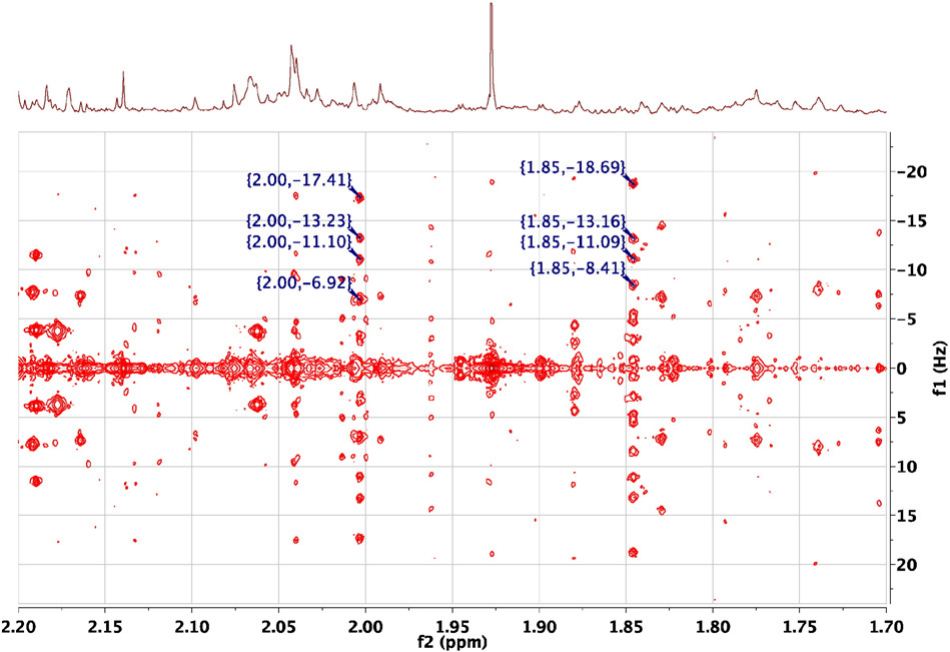
An expansion of the 600 MHz 2D ^1^H J-resolved NMR spectrum of the urine of a male, 30-week-old, FMO5 knockout mouse [61] in the region of the resonances from the C3 methylene hydrogens of 2S-hydroxyglutaric acid, displayed as a contour plot underneath the corresponding 1D ^1^H NMR spectrum. The spectrum is tilted by 45^0^, so that all the signals of each multiplet appear at the same chemical shift, and symmetrised. The peak picking allows a simple analysis of three of the four couplings that these hydrogens possess as 4.2, 6.3 and 10.5 Hz (2.003 ppm) and 5.5, 7.6 and 10.3 Hz (1.845 ppm). Note that these multiplets are invisible in the 1D ^1^H NMR spectrum.

An important use of 2D ^1^H JRES NMR spectra is to establish the magnitude of the coupling constants for the ^1^H NMR signals of particular hydrogen atoms. This can readily be done even when the metabolites are at low levels and the signals are difficult to see in the 1D ^1^H NMR spectra. For example, Fig. 9 shows an expansion from the 2D ^1^H JRES NMR spectrum of the urine of an FMO5 KO mouse at 30 weeks of age. The signals at 2.003 and 1.845 are from the two methylene hydrogens at C3 in 2S-hydroxyglutaric acid (HMDB00694). The chemical shifts of the two hydrogens are close to the values reported in the HMDB (1.985 and 1.825 respectively) but the assignment of the two hydrogens is much more secure if the coupling constants can also be shown to match. In this case the 1D ^1^H NMR FID of the authentic metabolite was downloaded from the HMDB and reprocessed. This showed that the line separations in the multiplets at 1.985 and 1.825 in the authentic metabolite were identical to those observed at 2.005 and 1.845 in the 2D ^1^H JRES NMR spectrum of the urine of an FMO5 KO mouse at 30 weeks age, thus helping confirm this metabolite identification.

### 3.3 2D ^1^H chemical shift correlation spectroscopy (COSY)

The 2D ^1^H chemical shift correlated spectroscopy (COSY) NMR experiment is a workhorse of metabonomics analyses for the identification of the metabolites in biological samples. Many variants of the 2D ^1^H COSY NMR experiment exist [35] but all variants provide information on which hydrogens are spin–spin coupled together, and this is vital for metabolite structure identification. The basic pulse sequence is: RD - 90^0^_H_ - t1 - 90^0^_H_ - FID, where RD is a relaxation delay. The first 90^0^ pulse excites all the nuclear spins: the second 90^0^ pulse causes coherence transfer between the magnetisations of hydrogens which are spin-coupled to one another. The reason for the importance of the COSY experiment can be best illustrated with an example. If we observe a methyl doublet signal in a urine sample at 1.34 ppm and that doublet signal has a coupling constant of 6.9 Hz, we could infer that that signal originated from lactic acid. However, if a 2D ^1^H COSY NMR spectrum of that urine sample indicated that the methyl doublet at 1.34 ppm was spin-coupled to a methyne proton at 4.13 ppm, that would be much stronger evidence that the methyl signal was indeed from lactic acid. The probability of known metabolite mis-identification decreases strongly with each successive connected spin matched to the corresponding signal in the spectrum of the authentic metabolite.

It is typical to run quick 2D ^1^H COSY NMR spectra with low digital resolution and often low sensitivity. This can be appropriate for the rapid analysis of pure chemical compounds but is not appropriate for metabonomics studies, as it results in the limited observation of hydrogen-to-hydrogen connectivities for major metabolites over 2-bonds or 3-bonds only. If the experiment is run at higher sensitivity and resolution, much more information can be gleaned, from a larger number of metabolites. Acquiring 2D COSY data at higher resolution can cost time, but this would not be done for every sample in a large metabonomics experiment. A high resolution COSY NMR spectrum would only be obtained on a handful of samples that are representative of the different groups in the study, with the express purpose of aiding metabolite identification.

**Fig. 10:**
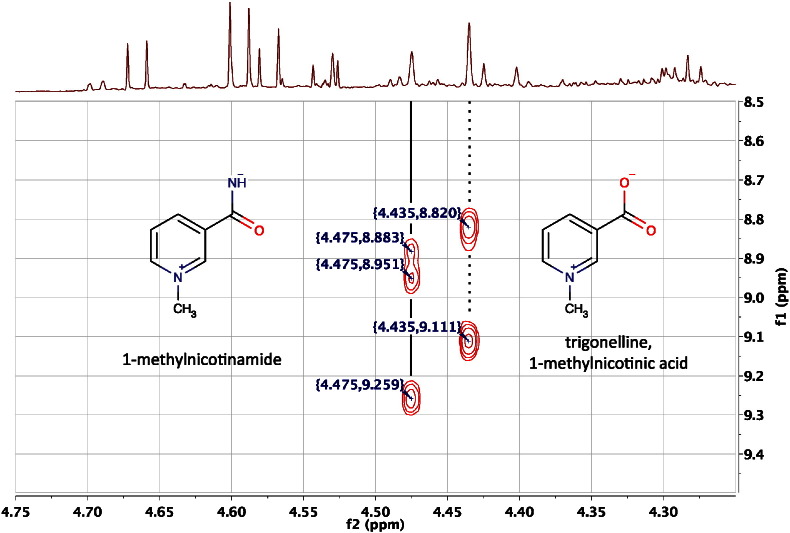
An expansion of the 600 MHz 2D ^1^H COSY NMR spectrum of the pooled urine of two male, 60-week-old, FMO5 knockout mice [61] in the region of the broad singlet methyl resonances from trigonelline at ca 4.435 and 1-methylnicotinamide ca 4.475 ppm, displayed as a contour plot underneath the corresponding resolution-enhanced 1D ^1^H NMR spectrum. Trigonelline displays cross-peaks due to long-range, 4-bond coupling from the methyl protons to the H2 (9.111) and H6 (8.820) protons ortho to the pyridinium nitrogen. 1-methylnicotinamide displays the same cross-peaks to H2 (9.259) and H6 (8.951), but in addition, displays a clear and remarkable cross-peak via six-bond coupling to H4 (8.883). The ability to connect the methyl shift with the pyridinium proton shifts in this way can assist metabolite identification enormously.

Using traditional methodology, a high resolution COSY spectrum might take several hours to acquire. For example, Fig. 10 shows an expansion of the 600 MHz 2D ^1^H COSY NMR spectrum of the pooled urine from two FMO5 KO mice [61] at 60 weeks age. This experiment was acquired with spectral widths in f1 and f2 of 9578 Hz, and 4096 points in the FID (t2) for 512 values of the evolution time (t1): the final spectrum was an 8192 by 2048 data matrix. The acquisition time was 0.428 s with a relaxation delay of 2 s, and 32 transients per increment of the evolution time, resulting in a total experiment time of just over 11 h, which is a significant investment of time. However, that additional time does allow correlations via small couplings over 4- to 6-bonds to be observed and these can be important for metabolite identification, as they enable connectivities to be established between parts of molecular structures isolated by so-called ‘spectroscopically silent centres’. These silent centres are atoms with no hydrogens attached or no non-exchanging hydrogens. These spectroscopically silent centres break up the chains of proton-to-proton connectivity in a metabolite that are important for metabolite identification by e.g. COSY NMR. In this case the silent centre is the pyridinium nitrogen, which has no hydrogens bonded to it.

**Fig. 11:**
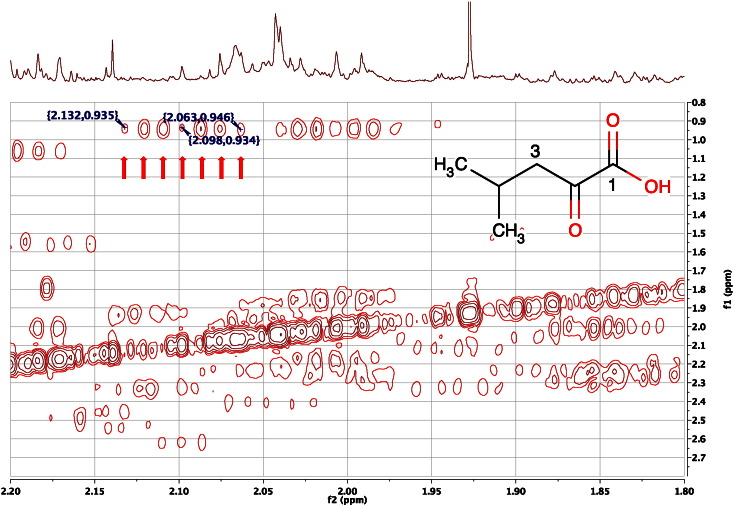
An expansion of the 600 MHz 2D ^1^H COSY NMR spectrum of the urine of a male, 30-week-old, FMO5 knockout mouse [61] highlighting with 7 arrows the cross-peak from the C4H proton of ketoleucine (structure inset) at 2.098 to the equivalent C5 and C6 methyl groups at 0.941 ppm. The signals from ketoleucine at 2.098 are not visible either in the 1D ^1^H NMR spectrum (top), or in the 2D ^1^H J-resolved NMR spectrum of the same sample (see Fig. 9) but the identification is confirmed from this high-resolution COSY spectrum. The seven cross peaks marked are the most intense peaks of the 9-line, pseudo-nonet, triplet of septets, the two outside lines of which are too weak to observe. See text for details.

When run at high resolution, the 2D ^1^H COSY NMR spectrum can also be used to identify the multiplicity of signals that are completely buried in the 1D ^1^H NMR spectrum, and even those that are buried in the 2D ^1^H JRES NMR spectrum. For example, the signal for the C4H methyne proton of ketoleucine at 2.098 ppm was invisible in the 1D ^1^H NMR spectrum (Fig. 11 top), or in the corresponding 2D ^1^H J-resolved NMR spectrum of the same sample (Fig. 9) but its identification is confirmed from the high-resolution COSY spectrum (Fig. 11).

The C4H peak of ketoleucine is a triplet of septets which appears as a pseudo-nonet, as the coupling from C4H to the C3H_2_ group (7.0 Hz) is very similar to the spin-coupling to the six equivalent methyl group protons (6.7 Hz). The two weak outside lines of the pseudo-nonet are weak and difficult to observe even in the authentic reference standard (BMRB, BMSE000383; HMDB00695 (caution the HMDB 1D ^1^H NMR was run at pH 3! [accessed 12 September 2015]). The high resolution COSY spectrum shown in Fig. 11 allowed the measurement of the frequency separation of highest and lowest frequency lines observed in the multiplet at 2.098 as 41.4 Hz, which corresponded well (COSY digital resolution in f2 = 0.73 Hz) to the separation in authentic material: 40.8 Hz in BMSE000383, thus providing further confidence for the assignment of this cross-peak.

The COSY experiment should always be run with good resolution in the FID (t2) as that resolution is essentially ‘free’. The increase in the acquisition time that this costs can be counterbalanced by a corresponding decrease in the relaxation delay between successive transients. Increasing the resolution across the second dimension, t1, does cost however, as does increasing the number of transients per value of the evolution time, and it is here that non-uniform sampling (NUS) methods and FAST NMR methods may lead to decreases in acquisition times in 2D NMR experiments for metabolite profiling in the future. Preliminary studies show promise and we await developments in this area with interest [64].

### 3.4 2D ^1^H total correlation spectroscopy (TOCSY)

The 2D ^1^H TOCSY NMR experiment, sometimes called ‘homonuclear Hartmann–Hahn spectroscopy’ (HOHAHA), is a relatively simple NMR experiment often used in conjunction with the COSY experiment to elucidate further structural information on small molecules of interest [65]. TOCSY provides similar information to a COSY experiment with regards to directly coupled hydrogens, but provides further structural information by identifying larger, interconnected groups of *indirectly* spin-coupled hydrogens.

In comparison to the COSY sequence, the second 90^°^_H_ pulse is replaced by a spin-lock field, applied for 10 s of milliseconds, which can be considered to behave like a series of 180° _H_ pulses. The spin-lock field eliminates chemical shifts during its application, but does not affect the scalar coupling. Due to the elimination of chemical shift differences in the spin-lock period, the spins are in a strong coupling regime, lose their individual identity and undergo magnetisation or coherence transfer. The magnetisation transfer that takes place is governed by the length of the spin-lock periods. Short spin-lock periods (20–100 ms) yield cross peaks for directly coupled spins. With longer spin-lock times (100–300 ms), coherence will be transferred more remotely down chains of spin-coupled hydrogens. Thus, if we have a spin system AMX, where A is coupled to M and M is coupled to X, but A is not coupled to X, two situations can arise in the TOCSY experiment. For short spin-lock periods, correlations will be seen between the chemical shifts of both A and M and of M and X. For longer spin-lock periods, cross-peaks will also be observed between A and X, even though they are not directly coupled.

**Fig. 12:**
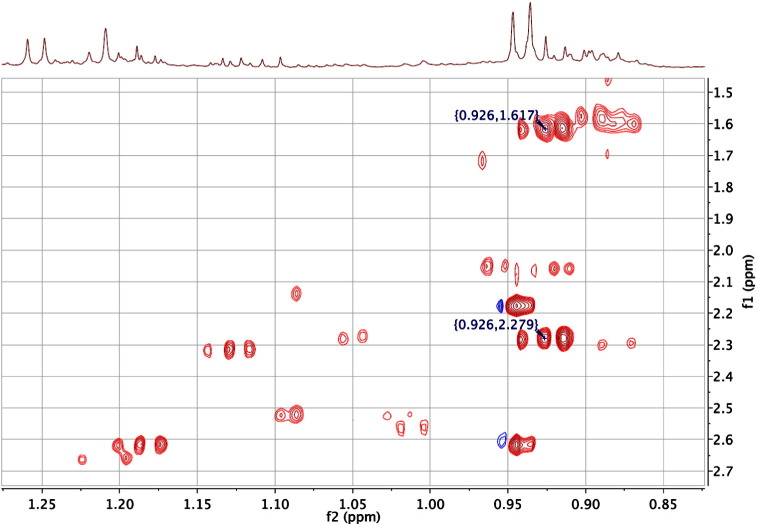
An expansion of the 600 MHz 2D ^1^H TOCSY NMR spectrum of the urine of a 30-week-old male C57BL/6 mouse. The cross peaks marked originate from the alkyl chain connectivities of *N*-butyrylglycine, from the terminal methyl group (C4). The cross peak marked at 0.926, 1.617 ppm represents a direct correlation from the C4 methyl protons to the adjacent C3 methylene group, equivalent to the cross-peak that would be observed in a 2D ^1^H COSY experiment. Additional metabolite identification information is provided in this TOCSY experiment however, with the cross-peak at 0.926, 2.279 ppm establishing a connection between the C4 methyl protons and the C2 methylene group, even though there is no observable coupling between them.

A good example of this can be shown in *N*-butyrylglycine (HMDB00808) which has an alkyl chain three carbons long. In a COSY experiment, the protons from the terminal C4-methyl group (0.926 ppm) would only have cross peak correlations with the adjacent C3-methylene protons (1.617 ppm). However, further structural information for *N*-butyrylglycine is provided (Fig. 12, see also Fig. 2, Fig. 7) when a cross peak is observed at the resonance of the remote C2-methylene protons (2.279 ppm).

A one-dimensional version of the TOCSY experiment is also available. The experiment involves the selective excitation of a signal followed immediately by the application of the spin-lock field to effect coherence transfer, essentially observing a slice of a 2D TOCSY. Chemical shift selective filter TOCSY (CSSF-TOCSY) uses excitation sculpting techniques with pulse field gradients to selectively excite overlapping proton signals with tiny chemical shift differences, enabling reliable extraction of coupling constants, important in metabolite identification.

### 3.5 2D ^13^C, ^1^H Heteronuclear Single Quantum Correlation (HSQC) NMR spectroscopy

The 2D ^13^C, ^1^H Heteronuclear Single Quantum Correlation (HSQC) NMR Spectroscopy experiment is another fundamental experiment for metabolite identification. The experiment operates by correlating the chemical shifts of hydrogens with the chemical shifts of carbon-13 nuclei to which they are directly attached via ^1^J_C,H_. The reason that this experiment is important is two-fold. Firstly, it introduces a completely new and orthogonal dimension beyond ^1^H NMR to obtain information on the structure of metabolites: that available from the C-13 NMR chemical shift. Secondly, the chemical shifts of the carbon-13 nucleus extend over about 220 ppm for most metabolites: this is ca 20 times the range of proton NMR chemical shifts (ca 11 ppm), and these ^13^C NMR shifts thus provide a much more sensitive response to minor changes in metabolite structure than does the ^1^H NMR chemical shift: see Section 2.2.

**Fig. 13:**
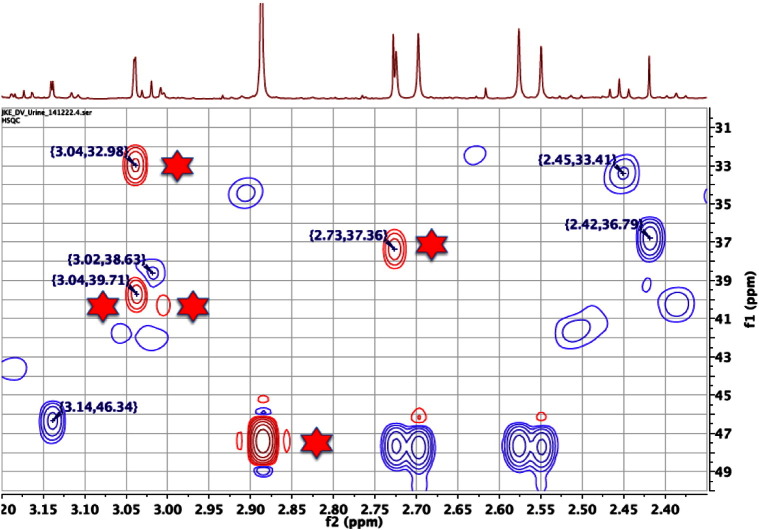
An expansion of the 600 MHz, multiplicity-edited, 2D 13C, ^1^H HSQC NMR spectrum of the pooled urine of 60-week-old, male, FMO5 knockout mice [61], displayed as a contour plot underneath the corresponding resolution-enhanced 1D ^1^H NMR spectrum. In this phase-sensitive plot, positive peaks are represented by red contours (asterisked) and negative peaks by blue contours (no asterisks). See text for further explanation.

Many variants of the 2D ^13^C, ^1^H HSQC NMR experiment are in current usage [35] and its successful execution does have some challenges. All variants of this experiment use ^1^H detection for high sensitivity and thus, not only must the enormous proton signals from water be suppressed, but also all of the signals from hydrogen atoms that are bound to carbon-12 nuclei, which is 99% of the hydrogens in each metabolite. Fortunately, the availability of high performance digital NMR spectrometers and gradient pulses has made the experiment routine. Indeed, new variants suitable for metabolite profiling in biofluids are now available that even provide carbon multiplicity editing as well. In these experiments, the 2D ^13^C, ^1^H HSQC NMR spectrum not only displays the cross-peaks due to ^1^J_C,H_ correlations, but also edits the cross-peaks in a phase-sensitive fashion so that the cross-peaks due to methyl (CH_3_) and methyne (CH) moieties are of opposite phase to those of methylene groups (CH_2_). This provides tremendous power for the assignment of signals in crowded regions of the ^1^H NMR spectra of a biofluid: see Fig. 13.

The multiplicity-edited, 2D 13C, ^1^H HSQC NMR spectrum in Fig. 13, readily distinguishes the red, positive cross-peaks (asterisked) arising from the methyl groups of creatinine (3.04, 32.98), creatine (3.04, 39.71) and dimethylamine (2.73, 37.36) from the blue, negative cross-peaks (no asterisks) due to the methylene groups in cis-aconitic acid (3.14, 46.34), 2-ketoglutaric acid (3.02, 38.63 and 2.45, 33.41) and succinic acid (2.42, 36.79). This experiment is a tremendous aid to the correct assignment of complex biofluid NMR spectra.

### 3.6 2D ^13^C, ^1^H Heteronuclear Multiple Bond Correlation (HMBC) NMR spectroscopy

**Fig. 14:**
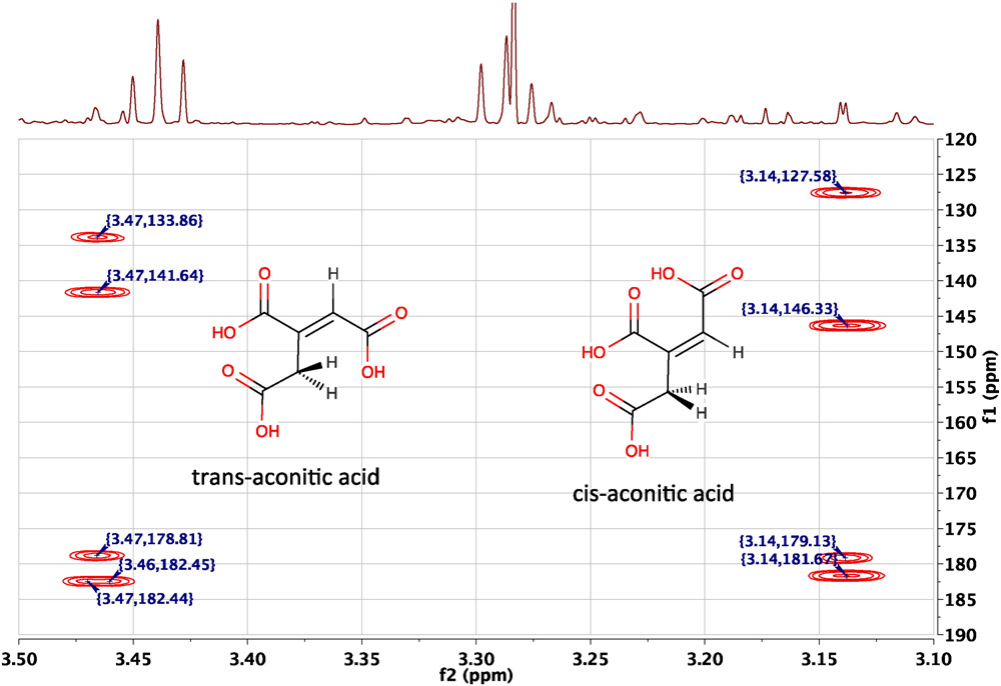
An expansion of the 600 MHz 2D ^13^C, ^1^H HMBC NMR spectrum of the pooled urine of male, 60-week-old, FMO5 knockout mice [61], displayed as a contour plot underneath the corresponding resolution-enhanced 1D ^1^H NMR spectrum in the region of the signals from the methylene protons of cis-aconitic acid (3.14) and trans-aconitic acid (3.47 ppm). The methylene protons display all four possible 2- and 3-bond hydrogen-to-carbon connectivities, to both adjacent carboxylic acid carbons (178.8, 182.4 ppm, trans- and 179.1 and 181.7 ppm, cis-isomer) plus connections to the quaternary and protonated olefinic carbons at 141.6 and 133.9 (trans-) and 146.3 and 127.6 ppm (cis-isomer), respectively, thus establishing connectivities between the two regions of protonated carbon structure isolated from each other by the quaternary olefinic carbon.

The 2D ^13^C, ^1^H Heteronuclear Multiple Correlation (HMBC) NMR Spectroscopy experiment [35] is another critical experiment in the identification of metabolites using NMR methods. The key reason for its importance is that it enables the establishing of connectivities between the parts of a metabolite's structure that are separated from one another by quaternary carbons or heteroatoms with no slow-exchanging, attached hydrogens. These are the so-called ‘spectroscopically silent centres’ mentioned earlier. The problem is that these silent centres interrupt the chains of proton-to-proton connectivity between regions of protonated carbons, resulting in isolated fragments of structure that may not be easy to piece together. To take a simple example, in the molecules cis- and trans-aconitic acid, the methylene moiety is separated from the olefinic proton by a quaternary carbon. In this case the HMBC experiment can help to connect the two fragments of protonated carbon structure together by establishing connectivities between hydrogens and carbon separated by two or three bonds (Fig. 14).

The HMBC experiment is critical for establishing connectivities between regions of protonated carbon structure when they are separated by quaternary carbons or heteroatoms. Although relatively insensitive, the HMBC experiment is sometimes the only way to obtain this information, if it is not available from alternatives such as high resolution COSY.

## 4 Metabolite identification

In this section we will bring together information obtained from 1D and 2D NMR experiments, together with information from metabolite databases and other sources to achieve metabolite identification and we will review methods for assessing the confidence in those metabolite identifications. There are essentially three strands to this activity: 1) the use of statistical methods to determine which NMR signals in a particular study are statistically significantly discriminating between groups of subjects in the study, or otherwise important, and therefore require identification and assignment, 2) the structure elucidation of novel metabolites, not previously described and 3) the structure confirmation of known metabolites. Some authors [28], [66] have described novel metabolites as unknown unknowns and known metabolites as known unknowns, but this language is confusing and unhelpful: we will retain the clear and simple distinction between novel metabolite structure *elucidation* and known metabolite structure *confirmation or identification*, that has been used in molecular structure studies by NMR spectroscopy for decades.

### 4.1 Identification of significant metabolites, or biomarkers, using multivariate statistics

The main objective in metabonomics is to extract relevant information from the large multivariate data sets. To this end pattern recognition (PR) and related multivariate statistical approaches can be used to discern meaningful patterns and identify metabolic signatures in the complex data sets that are of diagnostic or other classification value. A wide range of statistical methods is available today ranging from unsupervised methods, such as, principal component analysis (PCA), [67] or hierarchical clustering (HCA) [68], to supervised approaches like partial least squares (PLS) [69], partial least squares discriminant analysis (PLS-DA) and orthogonal partial least squares discriminant analysis (OPLS-DA) [70].

PCA is the most common technique in multivariate analysis that reduces the dimensionality of data and provides an unbiased overview of the variability in a dataset. In this approach samples are clustered based on their inherent similarity/dissimilarity with no prior knowledge of class membership. PCA represents most of the variance within a data set using a smaller set of variables, so-called principal components (PCs). Each PC is a weighted linear combination of the original variables, and each consecutive PC is orthogonal to the previous PC and describes the maximum additional variation in the data set that is not accounted for by the previous PCs. The results of a PCA are generally reported in terms of component scores, and loadings. In a scores plot, each point corresponds to a sample spectrum. Scores plots provide an overview of all samples and enable the visualisation of groupings, trends and outliers. A loadings plot illustrates which variables have the greatest contribution to the positioning of the samples on the scores plot and are therefore responsible for any observed clustering of samples. Since directions in the scores plot correspond to directions in the loadings plot, an examination of the loadings can explain spectral clustering observed on the scores plot [71], [72], [73]. Usually, PCA constitutes the first step in metabonomic data analysis and is commonly followed by supervised pattern recognition techniques. These methods use class information of the samples to maximise the separation between different groups of samples and detect the metabolic signatures that contribute to the classifications.

One commonly used supervised method is partial least squares, also known as projection to latent structures (PLS), which links a data matrix of predictors usually comprising spectral intensity values (an X matrix), to a matrix of responses containing quantitative values (a Y matrix). When the response matrix is categorical, i.e. the Y matrix contains sample class membership information, the application of PLS regression is called partial least squares-discriminant analysis (PLS-DA). PLS has also been used in combination with a pre-processing filter termed orthogonal signal correction (OSC), which excludes irrelevant parts of the data that are uncorrelated (orthogonal) with the response, often referred to as structured noise. This structured noise in the data set can be caused by analytical variation or by innate physiological variation (e.g. different diet, age, gender). Orthogonal partial least squares discriminant analysis (O-PLS-DA) has an advantage over the standard PLS because it filters the irrelevant variation and hence enhances the model interpretation and identification of important variables that are responsible for the observed classification [73], [74], [75]. Recently, a more advanced statistical technique, Statistical HOmogeneous Cluster SpectroscopY (SHOCSY), has been developed which can better address irrelevant variation in datasets and enhance the interpretation and predictive ability of the OPLS-DA model via the selection of ‘truly’ representative samples in each biological class [76].

In supervised techniques, loading weight, variable importance on projection (VIP) and regression coefficient plots are used to determine the most significant discriminating variables. Recently, a new approach has been introduced by Cloarec et al. that incorporates the back-transformed loading of an auto-scaled model with the respective weight of each variable in the same plot. The resulting loading plot created in this way has the same shape as that of a spectrum with colour-coded coefficients, according to statistical significance for each variable, which allows for easier interpretation of chemometric models [77].

Generally, supervised techniques are subject to overfitting, particularly in metabonomic studies where the number of variables is large and therefore the chance of false correlations is high. Proper model validation is therefore a key step to ensure model reliability and identification of true biomarkers. There are various validation methods including *k*-fold cross validation, permutation and test set validation. [78], [79], [80], [81] Cross validation is performed in most cases, especially when the number of samples is low. Here, the *k* subset of samples is iteratively left out and predicted back into the model until all samples have been used once. However, truly robust model validation is achieved by dividing the data into a training set and a test set. The training set is used to construct a model and the test set is used to assess the model performance.

### 4.2 Statistical Correlation Spectroscopy (STOCSY) for metabolite identification

**Fig. 15:**
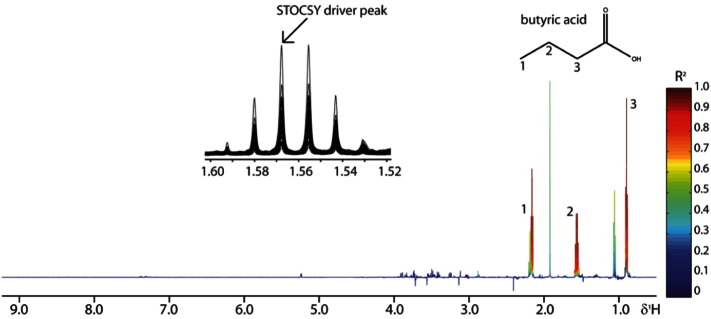
NMR plot following a STOCSY analysis on a set of faecal water ^1^H NMR spectra. The selected driver peak at 1.57 ppm was used to calculate the correlation matrix which reveals correlations ranging from 0 (low) to 1 (high). Two other resonances were revealed to have a positive correlation of 1, suggesting that they arise from the same molecule that was later identified as butyric acid.

Statistical Correlation Spectroscopy (STOCSY) follows the concept of two dimensional correlation spectroscopy which had originally been implemented in other spectroscopic techniques including fluorescence and Raman spectroscopies [82]. The development and adaptation of STOCSY in NMR spectra was initially performed by Cloarec et al. and is traditionally applied to one dimensional ^1^H NMR [74]. STOCSY takes advantage of the inherently linear relationship between intensity variables belonging to the same molecule in an NMR spectrum. It analyses the covariance of variables in a series of spectra and produces a correlation matrix, presented in the form of an NMR spectrum, which reveals the degree of correlation between each variable in the spectrum (either one-dimensional or two-dimensional; see Fig. 15). Depending on the strength of the correlation, correlated variables or resonances (consisting of many variables depending on the resolution) might belong to the same molecule (strong correlation) or molecules in the same metabolic pathway (weaker correlation). The correlation of each resonance, relative to the selected peak on which STOCSY is performed, is revealed by a colour scale which ranges from low correlation (typically 0) to high correlation (typically 1) [74]. In the field of metabonomics this technique is particularly useful in the analysis of complex mixtures, such as urine, where the identification of metabolites can be difficult due to the high density of resonances and potential overlapping [74].

It is important to clarify that the ability of STOCSY to detect the correct correlations is affected by the degree of overlap between resonances, as well as low concentrations. Significant overlapping with other peaks will distort the covariance of different resonances belonging to the same molecule in a spectrum, whilst resonances closer to the noise level are harder to analyse. Such deficiencies have led to the development of other techniques including SubseT Optimization by Reference Matching (STORM), which uses an iterative method to calculate the correlations and is better suited to dealing with potential overlaps or low concentrations [13].

### 4.3 Structure elucidation of novel metabolites

If a truly novel metabolite is identified in the course of a metabonomics study, then a full structure elucidation to the standard generally accepted for the identification of novel natural products [83] or novel drug degradation products [84] is required. This will usually entail the isolation and purification of the novel metabolite from the biofluid and a full structure elucidation, typically using NMR spectroscopy, MS, infrared spectroscopy and ultraviolet spectroscopy, and /or the synthesis of the metabolite for direct comparison with the data obtained from the biofluid.

### 4.4 Use of information from metabolite databases

Most metabolites observed by NMR spectroscopy in metabonomics studies will be known, and information on a proportion of these is available in various databases such as the Human Metabolome Database (HMDB) [45], the BioMagResBank (BMRB) [85] and the Birmingham Metabolite Library (BML) [86]. The HMDB is the largest repository of NMR and MS data on human metabolites that is currently available. As of September 4th 2015, the HMDB contained information on 41,993 metabolites. However, only 1381 of these metabolites have experimental NMR data, totalling 3186 NMR spectra. Thus, there are many metabolites for which it is not currently possible to access NMR data online. Databases such as the HMDB are valuable for four main reasons: 1) provide search facilities that allow the identification of known metabolites based on matches between user spectral data and database data on authentic metabolite samples, 2) provide interpreted 1D ^1^H and 2D NMR spectra (particularly 2D ^13^C, ^1^H HSQC spectra) of metabolites; 3) provide access to the raw free induction decay data for authentic metabolites for downloading, processing and comparison with user data on metabolites from biofluids and 4) provide metadata on the metabolites and links to other databases.

The 2D ^13^C, ^1^H HSQC search facility in the HMDB is particularly useful, as it uses both ^1^H and ^13^C NMR chemical shift information, and searches for matches between HSQC cross-peak coordinates input by a user and those of authentic metabolites in the database. This is a good place to start a metabolite identification exercise. The user must input the tolerances for the chemical shift differences between the user input values and database values: metabolites whose cross-peak coordinates are inside those tolerances will be returned as ‘candidate metabolites’. Chemical shifts will naturally be different between those of an authentic sample in water, D_2_O or phosphate buffer and those of the same metabolite in a biofluid such as urine or plasma, but generally ^1^H NMR chemical shifts should agree to +/− 0.03 ppm and ^13^C NMR chemical shifts to +/− 0.5 ppm. These differences will increase for ^1^H or ^13^C NMR chemical shifts in metabolites which can undergo tautomerism [87] of any kind and the shift differences may also be larger for nuclei close to ionisable groups in metabolites: both these features will be sensitive to environment.

When reviewing the candidate structures returned by the database that have HSQC features matching the user query, other information about the metabolite of interest will be used to discriminate the candidates. This information could include the multiplicity in the ^1^H NMR spectrum of the hydrogen giving rise to the HSQC signal, or connectivity information linking further elements of the metabolite structure from COSY or HMBC spectra. Of course, if the database search is done on just one HSQC cross-peak observed in the spectrum of the biofluid, ALL remaining HSQC cross-peaks in that metabolite should also be observable in the biofluid HSQC spectrum, and the absence of any of the expected HSQC cross-peaks would put a question mark over the identification of the metabolite. On the other hand, as seen above, even databases as large as the HMDB are incomplete and searches will return no candidate structures for known metabolites if: (i) the metabolite is not entered into the database, (ii) the metabolite is in the database but the relevant NMR data is not, or (iii) the metabolite is in the database but the relevant NMR data is not correctly entered.

A further caveat to the use of metabolite databases is that they are only as good as the quality of the data entered into them. Users must beware that errors of several type are present at a low level in current databases such as the HMDB, including incorrect samples, incorrect structures for the metabolites, impure samples and incorrect assignments. A good approach is to always download the original data and check it against expectations, and/or check the values given across more than one database where possible.

### 4.5 Prediction of NMR spectra of metabolites for structure confirmation

An ideal situation for the confident identification of known or novel metabolites would be to be able to predict their NMR spectra computationally without the need for access to authentic, real samples. In Section 2.2, we saw that ^13^C NMR chemical shifts could be predicted by hand for simple molecules. Accurate chemical shift prediction would allow the expansion of databases such as the HMDB to include all known metabolites and the confident identification of novel as well as known metabolites. At present, this approach is not generally possible. Software such as MNova [46] and Marvin [47] allows the prediction of ^1^H and ^13^C NMR spectra. In our experience, these approaches are useful and somewhat successful but may fail in cases where the metabolite structure is complex, or is complicated by tautomerism or multiple sites of ionisation, and the methodology cannot always compute these with confidence for the relevant biological matrix.

### 4.6 Biochemical transformation and in vitro fermentation of biofluids to aid metabolite identification

One successful approach to metabolite identification that is currently under-utilised is the biochemical transformation of unknown metabolite A in a biofluid to known metabolite B. This approach was used in the identification of para-cresol sulphate (PCS) as the key biomarker in human urine for the prediction of the metabolic fate of paracetamol [5]. Incubation of samples of the human urine containing PCS with a sulphatase enzyme led to the transformation of PCS to the known metabolite para-cresol, which was then readily identified in this first human pharmacometabonomics study.

**Fig. 16:**
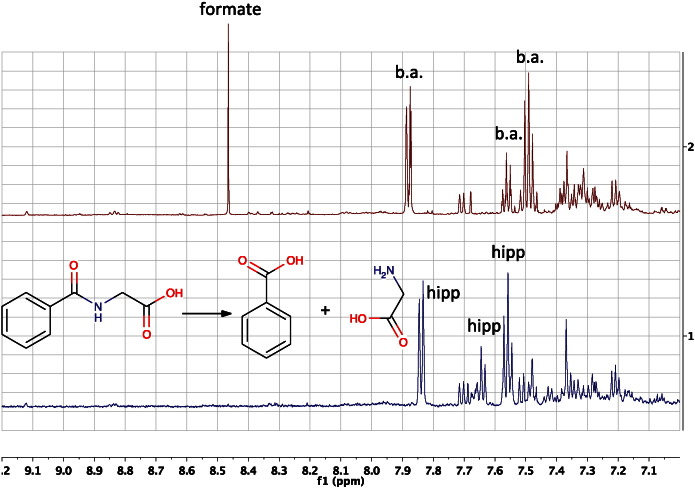
An expansion of the 600 MHz ^1^H NMR spectra of the urine of a male, 30-week-old, FMO5 knockout mouse [61]: 1) before bacterial fermentation and 2) after bacterial fermentation after leaving the sample at ambient temperature for several days. The bacterial fermentation caused many metabolic transformations including that of hippuric acid (hipp) to benzoic acid (b.a.) and glycine (3.57 ppm, not shown) and the formation of formate. The lower spectrum 1) prior to fermentation shows many signals including those from the ortho (7.84), para (7.64) and meta (7.56) protons of hippuric acid, whereas post-fermentation, spectrum 2) at top, shows corresponding signals from the ortho (7.88), para (7.56) and meta (7.49 ppm) protons of benzoic acid.

A more extreme and more random, but still useful, implementation of this approach can occur if biofluids are left at room temperature for extended periods of time. Biofluids such as mouse urine will quite likely have been in contact with faecal material and thereby be contaminated with bacteria from the animal's microbiome. It is standard practice in metabonomics studies to add a low concentration of an anti-bacterial agent such as sodium azide to animal urine samples to inhibit bacterial growth, but unless the concentration of azide is high, bacterial growth may still occur. This will cause in vitro fermentation in the urine and will transform large numbers of metabolites into different but related metabolite products. For instance, bacterial fermentation in a sample of urine from a male FMO5 KO mouse at age 30 weeks, led to the 100% conversion of hippuric acid (benzoylglycine) to benzoic acid and glycine (Fig. 16).

Compared with single enzymatic transformations, the in vitro fermentation approach is less specific. However, it is still a potentially useful tool to clarify metabolite identifications, by transforming unknown metabolites into known metabolites, or just to decrease crowding in a particular spectral region.

### 4.7 Confidence levels in known metabolite identification and confirmation of known metabolite identity

The Metabolomics Standards Initiative recognises 4 levels of known metabolite identification:

**Table:** 

**Level 1: Identified Compound:** A minimum of two independent and orthogonal data (such as retention time and mass spectrum) compared directly relative to an authentic reference standard
**Level 2: Putatively Annotated Compound**: Compound identified by analysis of spectral data and/or similarity to data in a public database but without direct comparison to a reference standard as for Level 1
**Level 3: Putatively Characterised Compound Class**: unidentified per se but the data available allows the metabolite to be placed in a compound class
**Level 4: Unknown Compound**: unidentified or unclassified but characterised by spectral data

These categorisations are somewhat vague in terms of the degree of fit between the data on the metabolite and that on the reference standard it is being compared to. They have not been widely adopted since their publication in 2007 [88], [89], and this has been commented upon recently [90]. Various modifications to the original categorisations have been suggested [91], [92] in order to improve them but with no general agreement on the way forward. A call to the community was made for engagement with this problem [91]. Encouragingly, a new, quantitative Bayesian method for annotation of metabolites in LC–MS experiments has recently emerged. [93] New quantitative NMR spectroscopy-based proposals have also been published [94] that reject the notion that known metabolite identification (as opposed to putative annotation (Level 2)) must always be based on a direct comparison of the experimental data on the metabolite in a biofluid with that of an authentic reference standard (Level 1 above). The new methods are based on the matching of information obtained experimentally from NMR studies of biofluids with that contained on authentic metabolites in databases such as the HMDB. These methods analyse the amount of matching 1D and 2D ^1^H NMR spectroscopic information obtained on each metabolite, relative to the number of carbon atoms or heavy atoms in the molecule. One promising new approach is called Metabolite Identification Carbon Efficiency (MICE) [94] and provides a logical, quantitative and systematic method for assessing confidence in known metabolite identification by NMR methods.

The use of metabolite database information, as opposed to information directly from the actual reference standards, to underpin metabolite identification is appropriate for NMR spectroscopy-based methods. In general, there is very good agreement between the chemical shifts of a metabolite in a buffered biological fluid such as urine and in a pure buffer solution of the same metabolite at the same pH. As mentioned in Section 4.4 above, generally, ^1^H NMR chemical shifts should agree to +/− 0.03 ppm and ^13^C NMR chemical shifts to +/− 0.5 ppm for most metabolites, although there will be cases of metabolites with greater chemical shift sensitivity, due to the arrangement of ionisable groups in their molecular structures, for instance, citric acid. There will be an even closer agreement between the chemical shifts of a reference standard run in similar buffers between one laboratory and the next. Therefore access to the NMR spectral data on a metabolite from a database such as HMDB is, in most cases, equivalent to having run the NMR spectrum of that material under the same conditions in the user laboratory. It must be stressed however, that all database data should be checked for quality and for matching to the expected structure. Mistakes in databases do occur: users should be aware. On the other hand, for MS-based metabonomics approaches, such as LC–MS or UPLC–MS, the use of authentic reference standards is more important, due to variations in metabolite retention times and peak intensities that can occur in these experiments, although new methods are making the metabolite annotations more secure [93].

The MICE method mentioned above is one of many new variants that can be used for the assessment of known metabolite identification confidence. In its recommended HSQC-level implementation [94], MICE counts and sums the number of bits of spectroscopic identification information obtained from ^1^H NMR chemical shifts, multiplicities, coupling constants, second-order flags (flag = 0 if metabolite signals are first order; flag = 1 if signals second-order [strict definition: additional lines present in the spectra]), 2D COSY cross-peaks and 2D HSQC cross peaks, for each metabolite, that *match* corresponding database values for the authentic metabolite. The MICE value is then obtained by dividing this information bit sum total by the number of carbon atoms in the metabolite. For example, the following signal features were observed for the metabolite ketoleucine, (4-methyl-2-oxopentanoic acid, HMDB00695, see Fig. 11 and structure below), in the 600 MHz ^1^H NMR spectra of the urine of a male, 30-week-old FMO5 knockout mouse [61]: a doublet (^3^J_H,H_ ca 7.0 Hz) for the H3 protons at 2.618 with a COSY to 2.098 (triplet of septets, H4), itself with a COSY to the equivalent methyl groups H5, and H6 at 0.941 (doublet, 6.7 Hz) and these with an HSQC to 24.5 ppm (C5, C6). Thus for this metabolite, we observed 3 ^1^H NMR chemical shifts, 3 multiplicities, two coupling constants, two COSY connectivities and one HSQC connectivity: a total of 11 pieces of information, all of which are a good match to the corresponding values in the HMDB. The guidelines for a good match are: within ± 0.03 ppm for ^1^H, and ± 0.5 ppm for ^13^C NMR shifts and ± 0.2 Hz for proton couplings. There are 6 carbon atoms in the molecule, so the Metabolite Identification Carbon Efficiency (MICE) = 11/6 = 1.8. MICE values of > 1 with a good match of spectral features to those of the standard in a database are considered confidently identified, as in this case.

**Figure:**
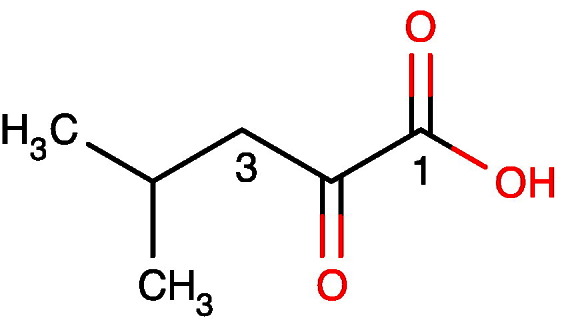

Even if a known metabolite is confidently identified by NMR spectroscopy using the MICE methodology, it can sometimes still be important to further confirm the identification, especially if the particular metabolite is an important biomarker. Three basic approaches are frequently used: 1) authentic metabolite spiking; 2) orthogonal analyses using MS-based approaches and 3) isolation or purification of the metabolite using chromatographic procedures. Metabolite spiking involves the addition of a small quantity of an authentic sample of the metabolite into the biofluid of interest and re-running the NMR spectrum. If the metabolite is present in the biofluid, then the signals of the spiked material should overlap exactly with those assigned to that metabolite in the original biofluid. For this experiment to work well, spectral resolution and lineshape must be optimal and it is best to spike in a quantity equalling between 25% and 50% of the material in the biofluid: too little material spiked can lead to uncertainty as to whether the signals of interest have increased in intensity: too much material spiked may swamp the signals and lead to uncertainty as to whether the spike signals match those of the metabolite of interest. Liquid chromatography–mass spectrometry (LC–MS) or ultra performance liquid chromatography MS (UPLC-MS) [29], [30] is often used as an orthogonal confirmatory technique for metabolites identified by NMR spectroscopy. This joint NMR and MS approach will provide the maximal confidence in the identification of known biomarkers that are particularly important. Isolation or purification procedures may use liquid chromatography, solid-phase extraction or liquid–liquid extraction methods [95], [96].

## 5 Conclusions and future thoughts

Metabonomics/metabolomics is undergoing a period of very rapid technology development and a huge increase in the number of applications, using mainly NMR spectroscopy or MS detection technologies. In this guide, we have focused upon the metabolite identification stage of a project using NMR spectroscopy-based detection of metabolites. Compared with MS, NMR spectroscopy is much less sensitive, but has the key advantages of better spectrometer stability, absence of spectrum quenching or enhancement phenomena, full quantitation of metabolites and the ability to use a huge range of the most powerful experiments for metabolite structure elucidation. NMR-detected metabonomics/metabolomics has been delivering answers to important questions in medicine, biology and other sciences for over 30 years and we confidently predict that it will continue to do so for decades more.

Many key advances in NMR spectroscopy-based metabonomics are emerging and these are expected have a significant impact on the utility of the technology. We can highlight the following: 1) the development of highly stable digital spectrometers producing spectra of unparalleled quality; 2) the development of probes with multiple receiver coils enabling the parallelisation of some data acquisition; [97] 3) the development of non-uniform sampling and spatially-encoded ‘ultrafast’ methods [64] of 2D NMR data acquisition, which hold out the prospect that in the future the default metabonomics experiment may be 2D COSY or 2D J-resolved rather than the current standard: 1D ^1^H NMR; 4) huge advances in the computational analysis of NMR data in methods derived from STOCSY that hold out the prospect of a systems biology analysis directly from the NMR data [98] and finally 5) the use of reliable, chilled, NMR sample automation systems which mean that large-scale experiments on hundreds or thousands of samples are feasible, enabling the advent of large-scale phenome analyses [12]. We await this future with excitement and much anticipation.

## Glossary of terms

**Table:** 

Term	Meaning
1D	one-dimensional
2D	two-dimensional
90^0^_H_	a 90 degree pulse to the ^1^H channel
180^0^_H_	a 180 degree pulse to the ^1^H channel
BML	Birmingham Metabolite Library
BMRB	BioMagResBank
CAWG	Chemical Analysis Working Group
COSY	COrrelation SpectroscopY
CPMG	Carr–Purcell–Meiboom–Gill
CSSF-TOCSY	Chemical Shift Selective Filter TOCSY
δ_H_	^1^H, hydrogen-1 or proton NMR chemical shift
δ_C_	^13^C or carbon-13 NMR chemical shift
FID	Free Induction Decay
FMO5	Flavin Mono-Oxygenase 5
GC–MS	Gas Chromatography–Mass Spectrometry
HCA	Hierarchical Cluster Analysis
HMBC	Heteronuclear Multiple Bond Correlation spectroscopy
HMDB	Human Metabolome DataBase
HOHAHA	HOmonuclear HArtman HAhn
HSQC	Heteronuclear Single Quantum Correlation spectroscopy
ID	identification
^3^J_H,H_	three-bond spin–spin coupling between two hydrogens
JRES	J-resolved spectroscopy
KO	gene Knock Out
LC–MS	Liquid Chromatography-Mass Spectrometry
MHz	MegaHertz = Hertz x 10^6^
MICE	Metabolite Identification Carbon Efficiency
MS	Mass Spectrometry
MSI	Metabolomics Standards Initiative
NOESY	nuclear Overhauser enhancement spectroscopy
NMR	Nuclear Magnetic Resonance
O-PLS-DA	Orthogonal-Partial Least Squares-Discriminant Analysis
PC	Principal Component
PCA	Principal Components Analysis
PLS	Partial Least Squares (Projection to Latent Structures)
RD	Relaxation Delay
SHOCSY	Statistical HOmogeneous Cluster SpectroscopY
STOCSY	Statistical TOtal Correlation SpectroscopY
STORM	SubseT Optimization by Reference Matching
t1	evolution time in a 2D NMR experiment
t2	the acquisition time over which the FID is measured
T_1_	spin–lattice relaxation time
T_2_*	real spin–spin relaxation time
TOCSY	TOtal Correlation SpectroscopY
TSP	sodium 3-(trimethylsilyl) propionate-2, 2, 3, 3-d4
UPLC–MS	Ultra-Performance Liquid Chromatography Mass Spectrometry
VIP	Variable Importance on Projection
